# Feasibility of endoscopic ultrasound‐guided hepaticogastrostomy using a novel long balloon catheter

**DOI:** 10.1002/deo2.70082

**Published:** 2025-02-14

**Authors:** Yuichi Takano, Naoki Tamai, Masataka Yamawaki, Jun Noda, Tetsushi Azami, Fumitaka Niiya, Naotaka Maruoka, Tatsuya Yamagami, Masatsugu Nagahama

**Affiliations:** ^1^ Department of Internal Medicine Division of Gastroenterology Showa University Fujigaoka Hospital Kanagawa Japan

**Keywords:** adverse events, balloon catheter, bile leakage, EUS‐HGS, tract dilation

## Abstract

**Objectives:**

Recently, a novel long balloon catheter for tract dilation in endoscopic ultrasound‐guided hepaticogastrostomy (EUS‐HGS) was developed. The balloon measures 6 cm in length, which enables one‐step tract dilation of the gastric wall, liver parenchyma, and bile duct wall, contributing to shorter procedure times and reduced bile leakage. This study investigated the feasibility of EUS‐HGS with this new balloon catheter.

**Methods:**

This retrospective study included consecutive cases in which EUS‐HGS was performed using a novel long balloon catheter (3 mm in diameter) for malignant distal biliary obstructions between February 2024 and October 2024. The patients' clinical background and procedural details were retrospectively examined using medical records. The primary outcome was technical success, defined as successful stent placement without additional dilation using devices other than the new balloon catheter. The secondary outcomes were clinical success and adverse events.

**Results:**

This study included 10 patients. The median age was 82.5 years, and there were seven males and three females. The median procedure time was 20 min. Technical success was achieved in 90% and clinical success was achieved in 100%. Regarding adverse events, one patient developed moderate cholecystitis, and percutaneous transhepatic gallbladder drainage was performed. No bile leakage, peritonitis, bleeding, or perforation was observed.

**Conclusion:**

The new long balloon catheter is an excellent device that can reliably dilate the whole tract with a single inflation. EUS‐HGS using a novel long balloon catheter is a feasible treatment option.

## INTRODUCTION

The first report of endoscopic ultrasound‐guided biliary drainage (EUS‐BD) was by Giovanini et al. in 2001.^1^ Today, EUS‐BD is widely performed and is an indispensable procedure, primarily as a rescue technique for cases of failed endoscopic retrograde cholangiopancreatography.^2^, ^3^, ^4^, ^5^ Moreover, there are also reports showing its potential as a technique for primary drainage.^6^, ^7^ There are various EUS‐BD techniques, such as EUS‐guided choledochoduodenostomy, EUS‐guided hepaticogastrostomy (EUS‐HGS), EUS‐guided rendezvous, EUS‐guided gallbladder drainage, EUS‐guided antegrade stenting (EUS‐AGS), and EUS‐HGS with antegrade stenting (EUS‐HGAS). The most common among these is EUS‐HGS, which can be performed in cases with surgically altered anatomy and duodenal obstruction.

In Japan, guidelines for EUS‐BD have been published, and the technique is becoming more mature.^8^ However, EUS‐HGS remains a highly difficult procedure that can cause serious adverse events. Notably, tract dilation before stent placement is an important step for the success of the procedure, but this is often difficult, especially for beginners. Smooth tract dilation is directly linked to the success of the procedure.

**FIGURE 1:**
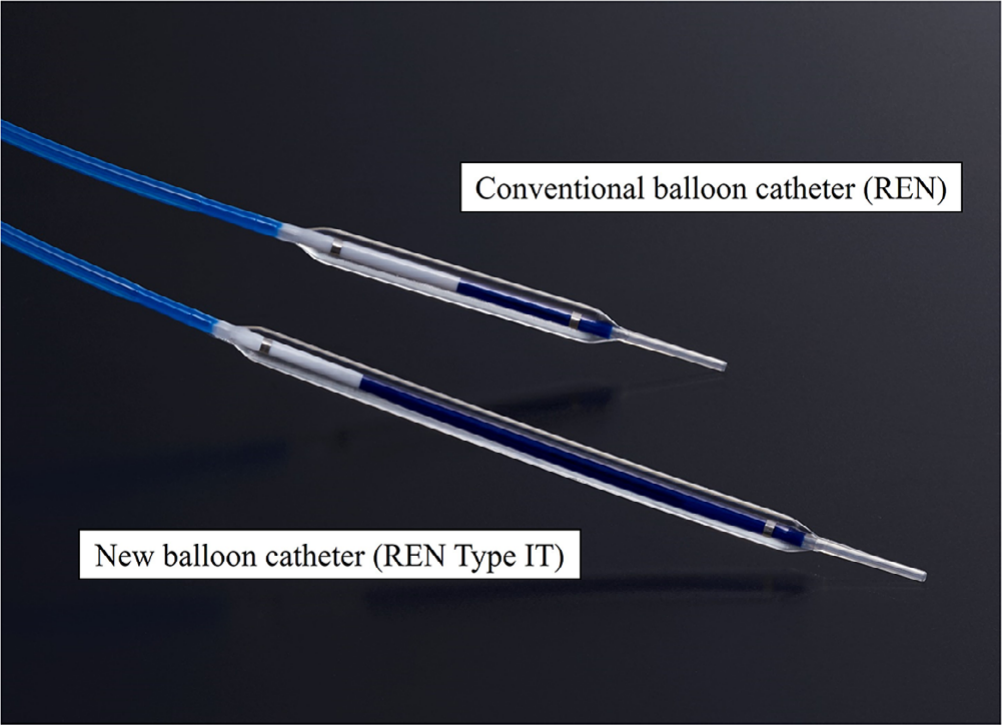
A novel balloon catheter (balloon diameter 3 or 4 mm, length 6 cm, REN biliary dilation catheter Type IT; Kakea Medix) has been developed for tract dilation in endoscopic ultrasound‐guided hepaticogastrostomy. The balloon length is 6 cm, which is longer than a conventional balloon catheter (2 or 3 cm). It enables one‐step tract dilation of the gastric wall, liver parenchyma, and bile duct wall, contributing to shorter procedure times and reduced bile leakage.

A novel long balloon catheter for EUS‐HGS (balloon diameter 3 or 4 mm, length 6 cm, REN biliary dilation catheter type IT; Kaneka Medix) was launched in 2024 (Figure 1).^9^ The tip of the catheter is only 3‐Fr and tapered, which is favorable for pushing. Compared to a conventional balloon catheter with a length of 2 or 3 cm, the balloon measures 6 cm in length, which enables one‐step tract dilation of the gastric wall, liver parenchyma, and bile duct wall, contributing to shorter procedure times and reduced bile leakage. This study investigated the feasibility of EUS‐HGS with this new balloon catheter.

## METHODS

This was a single‐center, retrospective study. This study was conducted with the approval of the Showa University Ethics Committee (Approval number: 2024‐203‐A) and in accordance with the tenets of the Declaration of Helsinki.

### Patients

This study included consecutive cases in which EUS‐HGS was performed using a new balloon catheter for malignant distal biliary obstructions between February 2024 and October 2024. The patients' clinical background and procedural details were retrospectively examined using medical records.

### Outcome measurement

The primary outcome was technical success, defined as “successful stent placement without additional dilation using devices other than the new balloon catheter.” The secondary outcomes were clinical success and adverse events, defined using the Tokyo criteria.^10^ Procedure time was defined as the time required from bile duct puncture to stent placement. The distance of the liver parenchyma was measured from the intrahepatic bile duct that was punctured to the periphery of the hepatic parenchyma on EUS imaging.

### The BD protocol at our hospital

Endoscopic transpapillary BD is the first choice in all cases. EUS‐HGS is performed in cases of duodenal obstruction, failed biliary cannulation, or difficult scope insertion due to surgically altered anatomy. When EUS‐HGS is unsuccessful, EUS‐CDS is considered technically feasible. In cases where EUS‐BD is unsuccessful, percutaneous transhepatic BD is performed.

### EUS‐HGS procedure

**FIGURE 2:**
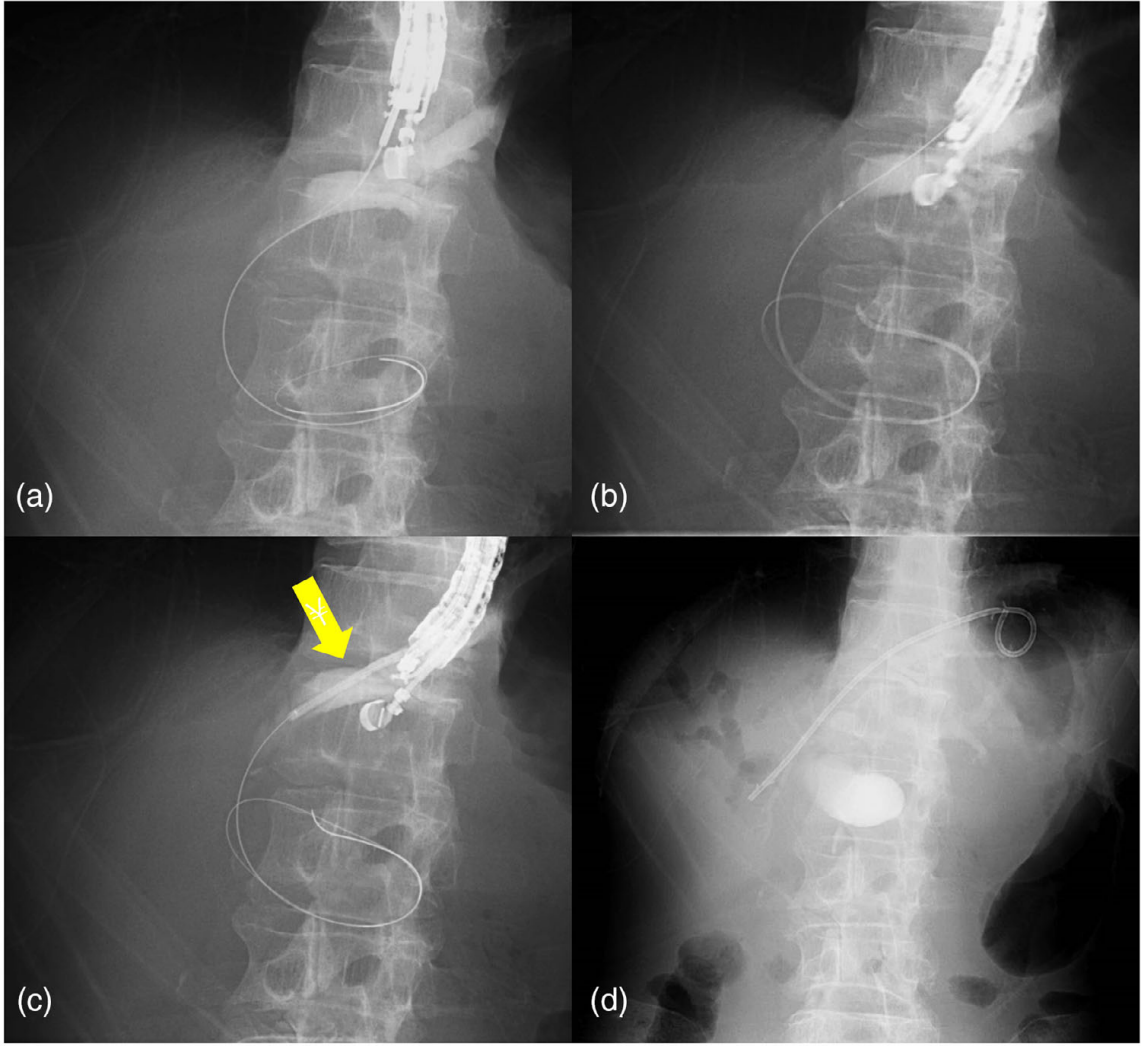
Tract dilation using a new balloon catheter in endoscopic ultrasound‐guided hepaticogastrostomy. (a) After puncturing B2, a 0.025‐inch guidewire was placed into the common bile duct. (b) A new long balloon catheter was inserted. (c) Tract dilation using the new balloon catheter. The inflated balloon is easily visible on the fluoroscopy screen (arrow). One‐step tract dilation of the gastric wall, liver parenchyma, and bile duct wall was achieved. (d) A 7‐Fr dedicated plastic stent for endoscopic untrasound‐guided hepaticogastrostomy was smoothly inserted.

An oblique viewing echoendoscope (GF‐UCT260; Olympus Medical Systems) was used in all cases. The observation device was a UE‐ME2 (Olympus Medical Systems). The intrahepatic bile duct was visualized transgastrically, and B2 or B3 was punctured using a 19G puncture needle (EZshot3; Olympus Medical Systems). After cholangiography, a 0.025‐inch guidewire (Visiglide2; Olympus Medical Systems) was inserted. A catheter (SHOREN; Kaneka Medix) was inserted into the bile duct, and the bile was aspirated as much as possible. If the insertion of the catheter was impossible, it was inserted after tract dilation and bile was aspirated. The tract was dilated using a new balloon catheter with a diameter of 3 mm and a length of 6 cm (REN biliary dilation catheter Type IT, Kaneka Medix). Finally, a 7‐Fr plastic stent dedicated to EUS‐HGS (Through & Pass Type IT; Gadelius) was placed to complete the procedure (Figure 2). EUS‐AGS was not performed.

## RESULTS

This study included 10 patients whose clinical backgrounds are described in Table 1. The median age was 82.5 years, and there were seven males and three females. The primary diseases were pancreatic cancer (*n* = 6), ampulla of Vater cancer (*n* = 2), colorectal cancer (*n* = 1), and renal cancer (*n* = 1).

**TABLE 1:** Clinical background of the cases.

Age, median (range)	82.5 (54–92)
Sex male/female, *n*	7/3
Primary disease, *n*	
Pancreatic cancer	6
Vater ampulla's cancer	2
Recal cell carcinoma	1
Colorectal cancer	1
Serum total bilirubin, median (mg/dL), median (range)	9.0 (2.3–21.9)
Cases with ascites, *n*	4
Previously placed transpapillary stent, *n*	1

Table 2 describes the procedural details of EUS‐HGS. There were no cases in which EUS‐HGS was performed as the primary drainage. The indications for EUS‐HGS were duodenal obstruction in six cases, biliary cannulation failure in three cases, and recurrent cholangitis after endoscopic transpapillary BD in one case. The median procedure time was 20 min, while B2 and B3 puncture, respectively, was performed in six and four cases. The median X‐ray angle between the puncture needle and the bile duct was 32.5 degrees. The median length of the liver parenchyma at the puncture site was 2.6 cm.

**TABLE 2:** Details of endoscopic ultrasound‐guided hepaticogastrostomy.

Indication of EUS‐HGS, *n*	
Duodenal obstruction	6
Biliary cannulation failure	3
Recurrent cholangitis after endoscopic transpapillary biliary drainage	1
Procedure time (min), median (range)	20 (13–45)
Puncture site, *n*	
B2	6
B3	4
Angle between puncture needle and bile duct (degrees), median (range)	32.5 (12–90)
Liver parenchyma distance (cm), median (range)	2.6 (1.5–3.1)

Abbreviation: EUS‐HGS, endoscopic ultrasound‐guided hepaticogastrostomy.

The outcomes of the EUS‐HGS are shown in Table 3. Technical success was achieved in 90% (9/10). In one case, a plastic stent could not be inserted after tract dilation, and additional dilation with a 7‐Fr bougie dilator (ES dilator; Zeon Medical) was needed before the stent was successfully placed. Regarding adverse events, one patient developed moderate cholecystitis, and percutaneous transhepatic gallbladder drainage was performed. No bile leakage, peritonitis, bleeding, or perforation was observed. Clinical success was achieved in 100%.

**TABLE 3:** Outcomes of the procedure.

Technical success, *n* (%)	9 (90)
Clinical success, *n* (%)	10 (100)
Adverse events, *n* (%)	1 (10)
Bile leakage, *n*	0
Peritonitis, *n*	0
Perforation, *n*	0
Bleeding, *n*	0
Cholecystitis, *n*	1
Pancreatitis, *n*	0
Liver abscess, *n*	0
Stent migration, *n*	0

## DISCUSSION

The EUS‐HGS procedure consists of four steps: (1) puncturing the intrahepatic bile duct, (2) manipulating a guidewire into the bile duct, (3) tract dilation, and (4) stent placement. Of note, tract dilation is an essential step for the success of the procedure.

Various dilation devices are currently available; these are classified into mechanical dilators and electrocautery dilators. Mechanical dilators are further classified into bougie dilators and balloon dilators. In previous reports, tract dilation with various devices has achieved favorable success rates of over 90%.^11^, ^12^, ^13^, ^14^, ^15^ Although tract dilation may be easier with electrocautery dilators, there is a risk of perforation and bleeding due to damage to the surrounding tissues and blood vessels. Honjo et al. reported that bleeding was significantly more frequent with electrocautery dilators versus mechanical dilators (18.1% vs. 0%).^14^ Accordingly, the Japanese EUS‐BD guidelines recommend the use of mechanical dilators as the first choice, and electrocautery dilators are recommended only when mechanical dilators are unsuccessful.^8^

In recent years, a 7‐Fr drill‐type dilator has been developed based on a new concept.^16^, ^17^ By rotating it clockwise, smooth tract dilation is possible without pushing the device too hard. Its usefulness has been shown not only in EUS‐HGS but also in EUS‐guided pancreatic duct drainage.^18^ Cases with stiff bile duct walls and liver parenchyma are good candidates for this drill‐type dilator. Hattori et al. reported a success rate of 100% (19/19) for drill‐type dilator dilation in EUS‐HGS, although 73.7% (14/19) of cases required additional dilation with a balloon catheter (4 mm) to insert an 8.5‐Fr metal stent delivery system. On the other hand, out of 30 cases wherein dilation was performed with a balloon catheter from the beginning, none required additional dilation.^19^ Thus, although the drill‐type dilator is a device with excellent pushability, the dilation may not be sufficient for an 8.5‐Fr delivery system.

Ogura et al. conducted a unique study on the dilation force of various devices, reporting that the 4‐mm diameter balloon catheter had the strongest dilation force among the mechanical dilators.^20^ This strong dilation force of the balloon catheter can enable smooth stent insertion.

In this study, the technical success rate, defined as successful stent placement without additional dilation using devices other than the new balloon catheter, was 90% (9/10). Additional dilation with a 7‐Fr bougie dilator was needed in one case since it was impossible to insert a plastic stent after the initial dilation. In that case, the puncture was performed from B3, which may have made it difficult to transmit force to the stent, thus causing the need for additional dilation. In all cases, it was possible to dilate the gastric wall, liver parenchyma, and bile duct wall with a single inflation. Therefore, the new balloon catheter is an ideal dilation device for EUS‐HGS.

The balloon used in this study had a diameter of 3 mm, which can theoretically expand to 9 Fr. When placing a 7‐Fr (2.1 mm) plastic stent, there is a possibility of overdilation. Mukai et al. pointed out the risk of bile leakage when placing a 7‐Fr plastic stent. Thus, they proposed a strategy of using a 7 Fr bougie dilator as the first choice when placing a plastic stent and using a new balloon catheter when the puncture angle is steep or when metal stent placement is planned.^9^

No cases of bile leakage or peritonitis were observed in this study. Ishiwatari et al. reported that aspirating 10 cc or more of bile during EUS‐HGS can prevent adverse events.^21^ In this study, bile aspiration was performed in all cases, which may have contributed to the reduction of adverse events, including bile leakage. Yamamoto et al. also reported that having liver parenchyma measuring less than 2.5 cm at the puncture site was an independent risk factor for bile peritonitis.^22^ The liver parenchyma may have a natural tamponade function. In this study, the median length of the liver parenchyma at the puncture site was 2.6 cm; the tamponade effect of the liver parenchyma could have prevented bile leakage.

The plastic stent used for EUS‐HGS used in this study was first reported by Umeda et al. in 2015.^23^ They reported 23 cases of EUS‐HGS in which a dedicated 8‐Fr plastic stent was placed. Among these, 30.4% (7/23) involved dilating the tract with a balloon catheter (4 mm). Adverse events were noted in 16.6% (4/23; mild abdominal pain, *n* = 3; moderate bleeding, *n* = 1), and no obvious bile leakage was observed. Thus, the plastic stent dedicated to EUS‐HGS may be highly safe and useful.

A serious adverse event of self‐expandable metallic stent placement is the migration of the stent into the abdominal cavity, which should be avoided at all costs, as it can be fatal.^24^ Plastic stents have the following advantages: 1) intraperitoneal migration of the stent is extremely rare, 2) focal cholangitis and liver abscesses due to bile duct branch obstruction are unlikely to occur, and 3) they are inexpensive. For these reasons, we place plastic stents as the first choice for EUS‐HGS.

After tract dilation, there is always a risk of bile leakage into the peritoneal cavity, which increases after multiple device exchanges and prolonged procedure time. Thus, it is desirable to place a stent as soon as possible after tract dilation. Yagi et al. investigated 38 cases of EUS‐HGS using a covered self‐expandable metallic stent (8.5‐Fr delivery system) and compared the outcomes of the balloon dilator (4–6 mm) versus the bougie dilator.^25^ No cases required additional dilatation in the balloon dilator group (0/17), but this was required in 28.6% (6/21) of cases in the bougie dilator group. Peritonitis was significantly less frequent in the balloon dilator group versus the bougie dilator group (5.9% vs. 14.3%) In particular, peritonitis was observed in 33.3% (2/6) of cases that required additional dilatation. A prolonged procedure time due to the additional dilation may cause bile leakage.

The balloon length of the conventional REN is short (2 or 3 cm), and multiple dilations are usually required during EUS‐HGS. Multiple dilations increase procedure time and the risk of bile leakage. In addition, it can be difficult for beginners to place the short balloon in the appropriate position. The novel balloon catheter has a 6 cm balloon, which is longer than the conventional REN. Therefore, even relatively long tract in EUS‐HGS can be dilated with a single inflation, contributing to shorter procedure time and reduced bile leakage. We believe this is the greatest advantage of the novel balloon catheter.

It has been reported that EUS‐HGAS can achieve a longer TRBO than EUS‐HGS alone.^26^ However, it is sometimes technically difficult to break through biliary stricture with the guidewire during EUS‐HGAS, and the procedure time may be longer. There is also concern about an increase in adverse events (especially bile leakage) due to prolonged procedure times. In the initial EUS‐BD, we give top priority to creating a reliable fistula (hepaticogastrostomy) without adverse events and simply placing a plastic stent. If stent obstruction occurs within a short period of time, we consider replacing it with a metal stent or adding EUS‐HGAS.

The limitations of this study include its single‐center, retrospective design and small number of cases. In addition, because our hospital has not used conventional REN for EUS‐HGS, we could not directly compare the outcomes between conventional REN and REN Type‐IT. Future prospective, comparative studies with a large number of cases are desirable.

In conclusion, the new long balloon catheter in EUS‐HGS is an excellent device that can reliably dilate the whole tract with a single inflation. EUS‐HGS using a novel balloon dilator is a feasible treatment option.

## CONFLICT OF INTEREST STATEMENT

None.

## ETHICS STATEMENT

Approval of the research protocol by an Institutional Reviewer Board. This is a single‐center, retrospective, and observational study that was approved by the ethics committee of Showa University (Approval number: 2024‐203‐A).

## PATIENT CONSENT STATEMENT

N/A

## CLINICAL TRIAL REGISTRATION

N/A
